# Vitamin D Level in Blood Serum and Its Connection with Helicobacter pylori Infection: A Systematic Review

**DOI:** 10.7759/cureus.102451

**Published:** 2026-01-28

**Authors:** Yasmine A Mohammed, Mariam R Elkhayat, Osama M El-Asheer, Medhat A Saleh, Nermeen A Gendy, Aml E Metwally, Amira T Anees, Mervat A Melek, Usama M Hasan, Doaa M Genena

**Affiliations:** 1 Pharmacology and Therapeutics, Department of Pharmacology, Faculty of Medicine, Assuit University, Assiut, EGY; 2 Nutrition, Public Health and Community Medicine, Faculty of Medicine, Assiut Unversity, Assiut, EGY; 3 Pediatric Medicine, Faculty of Medicine, Assiut University, Assiut, EGY; 4 Nutrition, Public Health and Community Medicine, Assiut University, Faculty of Medicine, Assiut, EGY; 5 Pharmacology and Therapeutics, Department of Pharmacology, Faculty of Medicine, Assiut, EGY; 6 Nutrition, Faculty of Medicine, Sohag University, Sohag, EGY; 7 Anaesthesiology, Faculty of Medicine, Assiut University, Assiut, EGY; 8 Family Medicine, Faculty of Medicine, Assiut University, Assiut, EGY; 9 Public Health, Medical Research Institute, University of Alexandria, Alexandria, EGY

**Keywords:** 25(oh)d, helicobacter pylori, peptic ulcer, prevention of infections, vitamin d deficiency

## Abstract

*Helicobacter pylori* (*H. pylori*) is widely recognized for its role in gastric pathologies, including ulcerative disease and malignant transformation of the gastric mucosa. Lower serum concentrations of 25-hydroxyvitamin D may impair immune responses, potentially influencing colonization dynamics and persistence of *H. pylori* infection.

A systematic review was conducted to critically evaluate the current evidence on the association between serum vitamin D levels and *H. pylori* infection. PubMed, Scopus, Cochrane Library, and Web of Science databases were comprehensively searched for relevant articles from their inception until May 28, 2025. Twenty observational studies, including 167,903 cases, were included in the review. Our results suggest a possible link between vitamin D deficiency and the risk of *H. pylori *infection, as the *H. pylori*-positive population was more likely to have lower vitamin D levels than the negative population. However, we could not confirm causality. In addition, vitamin D receptor (VDR) gene polymorphism, particularly rs2228570 (FokI), rs7975232 (ApaI), and rs1544410 (BsmI), as well as the TLR4 gene (rs4986790 and rs4986791), were significantly associated with *H. pylori* infection and vitamin D status. Our findings provide a strong base for further research into vitamin D’s role in gastric immunity and its utility as a modifiable risk factor in managing *H. pylori* infection.

## Introduction and background

*Helicobacter pylori* (*H. pylori*) is a Gram-negative, spiral-shaped bacterium that colonizes the human gastric mucosa and affects over half of the global population [1]. It is recognized as a Group 1 carcinogen by the World Health Organization. It is strongly implicated in the pathogenesis of chronic gastritis, peptic ulcer disease, mucosa-associated lymphoid tissue lymphoma, and gastric adenocarcinoma [2]. The burden of *H. pylori* infection disproportionately affects populations in low- and middle-income countries, where socioeconomic factors such as overcrowding, poor sanitation, and limited access to healthcare contribute to increased transmission and reinfection rates [3,4].

Vitamin D plays a vital role in innate and adaptive immunity, influencing monocyte and cytokine regulation and macrophage differentiation, and regulating antimicrobial peptide expression such as cathelicidin and β-defensin [5]. Moreover, its receptor, the vitamin D receptor (VDR), is expressed in immune cells, gastric epithelium, and hepatic tissue, suggesting its involvement in mucosal defence and inflammatory modulation [6-8]. The immunological relevance of vitamin D in infectious disease contexts has gained traction, with emerging evidence suggesting a potential inverse relationship between serum 25-hydroxyvitamin D levels and increased susceptibility to bacterial infections, including *H. pylori*, reduced eradication success, and enhanced inflammatory responses [5,9].

Recent epidemiological and molecular studies have substantiated this link. A retrospective study in Lebanese adults demonstrated significantly lower serum 25(OH)D levels in *H. pylori*-infected individuals compared to uninfected controls (p < .001), with deficiency strongly associated with infection risk [10]. Adding to this narrative, a recent meta-analysis synthesized findings from 12 observational studies encompassing diverse geographic regions. The pooled standardized mean difference (SMD) indicated that *H. pylori*-positive individuals had serum vitamin D levels 0.76 ng/ml lower than uninfected controls, while patients with successful eradication exhibited levels 1.53 ng/ml higher than those with eradication failure. Despite heterogeneity in study design and population characteristics, sensitivity analyses and publication bias assessments reinforced the robustness of these estimates [11]. Collectively, these findings suggest a multifactorial interplay between vitamin D metabolism, genetic predisposition, and host immune responses in determining the susceptibility and clinical manifestations of *H. pylori* infection. Elucidating these relationships may enhance risk stratification and inform novel adjunctive strategies in *H. pylori* management. Existing studies vary in inclusion criteria, vitamin D thresholds, and eradication protocols, necessitating a more rigorous synthesis. Furthermore, mechanistic pathways, such as VDR-mediated transcriptional regulation, autophagic clearance of *H. pylori*, and vitamin D's effect on gastric mucosal integrity, remain underexplored in clinical contexts.

In light of these gaps, this systematic review aims to critically evaluate the current evidence on the association between serum vitamin D levels and *H. pylori* infection. By integrating observational data, mechanistic insights, and geographic variability, we seek to determine whether vitamin D serves as a modifiable biomarker or adjunctive therapeutic target in the management of *H. pylori*. This investigation holds relevance not only for gastroenterology but also for public health strategies in regions burdened by both hypovitaminosis D and high *H. pylori* prevalence.

## Review

Methods

This systematic review followed the Preferred Reporting Items for Systematic Reviews and Meta-Analyses (PRISMA) checklist guidelines [12].

Literature Search

PubMed, Scopus, Cochrane Library, and Web of Science databases were comprehensively searched for relevant articles from inception until May 28, 2025. We used a combination of the following terms: “*Helicobacter pylori*” OR “*H. pylori*” AND “Vitamin D” OR “Vit. D”.

Eligibility Criteria and Procedure for Study Selection

Our study question was, “Is there any connection between *H. pylori* infection and vitamin D levels in blood serum?” According to PECOS, P (population) refers to the general population with no restriction on age, sex, or race; E (exposure) refers to *H. pylori* infection; C (comparator) refers to individuals with no *H. pylori* infection; O (outcome) refers to serum vitamin D levels; and S (study design) refers to observational studies (cohort, case-control, and cross-sectional). There were no restrictions regarding the publication date or country. The exclusion criteria included any editorial, case reports, case series, reviews, non-English studies, and studies with abstracts only available.

The Rayyan website (http://rayyan.qcri.org) [13] was used to combine all the retrieved records from the databases, which are free online, and throughout the entire screening process, which consisted of two steps: title and abstract screening, followed by full-text screening. At least two authors did each step, and blinding was maintained throughout the process using the blinding tool on the Rayyan website. Any disagreements were solved by discussion with an experienced author.

Data Extraction

We used a pre-formed Microsoft Excel (Microsoft Corporation, Redmond, WA) spreadsheet to extract the population baseline characteristics, study outcomes, summary of the studies, and data for quality assessment. Data for baseline characteristics and study summary included study ID, study design, study groups, and number in each group, age, male sex, BMI, alcohol and smoking status, presence of other comorbidities, the method used to diagnose *H. pylori* infection, and the conclusion of each study. Outcomes included serum vitamin D levels and genetic polymorphism.

Quality Assessment

The Newcastle-Ottawa Scale (NOS) was used to assess the risk of bias of the included studies [14]. It consists of three main domains. The first domain assesses participant selection (a maximum of five stars for cross-sectional and four for cohort studies), the second evaluates the comparability between the different study groups (a maximum of two stars), and the last domain assesses the outcomes and statistical analysis (a maximum of three stars). Hence, the maximum score was 10 stars for cross-sectional and nine for cohort studies. The studies were judged to have a low risk (high quality) if the score was greater than seven stars, a moderate risk (medium quality) if the score was between four and seven stars, and to have a high risk of bias (low quality) if the score was less than four stars.

Results

Search Results

**Figure 1:**
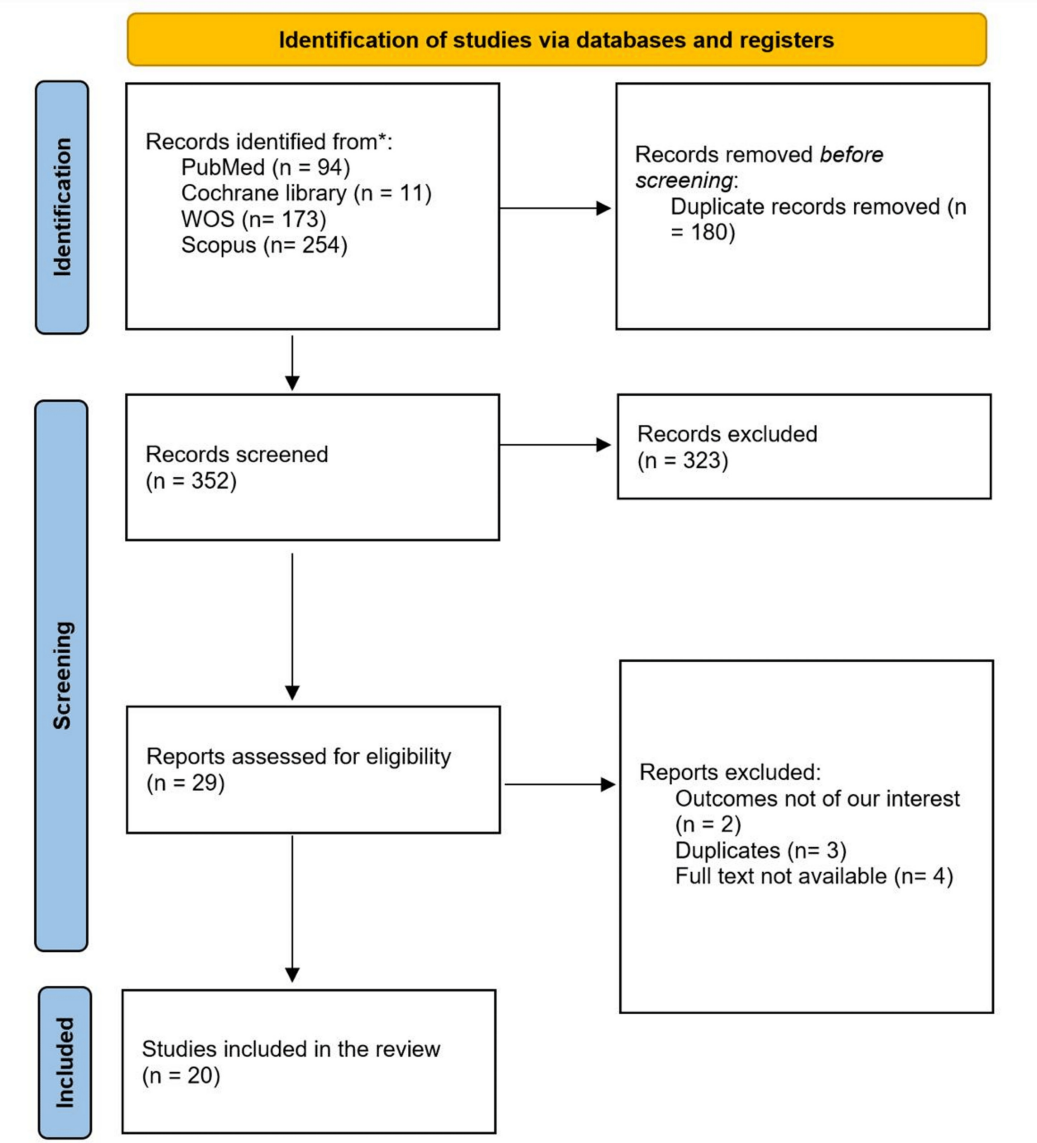
The PRISMA figure showing the steps to choose the studies for systematic review PRISMA: Preferred Reporting Items for Systematic Reviews and Meta-Analyses; WOS: Web of Science

The flow diagram of the screening process and studies' inclusion is shown in Figure 1. We retrieved 532 results from different databases; 180 were excluded as duplicates. Out of the remaining 352 results, 323 were excluded for not meeting the inclusion criteria, leaving 29 studies that went through the full-text screening phase. After excluding two studies for not meeting our inclusion criteria, three duplicates, and four studies because of the unavailability of their full texts, 20 studies were included in our systematic review.

Study Characteristics and Baseline Data

Out of the 20 [10,15-33] included studies in our systematic review, nine were observational cohort studies (four prospective and five retrospective studies), and 11 were cross-sectional studies. The included studies addressed about 167,903 cases, with a male percentage of 50.6% (84,970 cases). The mean age of the included population ranged between 1.04 (0.69) years old and 75.48 (5.85) years old. The diagnostic tool for *H. pylori* infection varied between the studies, including histopathological examination of gastric biopsy (nine studies), which is the most common tool. Other diagnostic methods included the urea breath test, serum antibodies, or stool examination. Other coexisting conditions and baseline characteristics are represented in Table 1.

**Table 1:** Summary and baseline characteristics of the included studies. M ±SD: mean ±standard deviation; N (%): number (percentage); NA: not available; a: *H. pylori* (+) = 335 (60.1); b: H. pylori (+) = 791 (50.8%). *H. pylori*: *Helicobacter pylori; *VDR:* *vitamin D receptor; NA: not available

ID	Study design	Study groups	Number	Age in years, M ±SD	Male sex, N (%)	Other coexisting conditions, N (%)		BMI					Diagnostic method	Conculosion
								M ±SD	N (%)					
						Renal disease	Liver disease		< 18.5	18.5–24.9	25–29.9	≥ 30		
Bahsi et al., 2020 [15]	Cross-sectional	*H. pylori* (+)	32	75.48 ±5.85	59 (58.4)	NA	NA	27.76 ±5.31	NA	NA	NA	NA	Histopathological examination of the gastric biopsy	Patients with sarcopenia and *H. pylori* infection had significantly high levels of serum vitamin D
		*H. pylori* (-)	69			NA	NA		NA	NA	NA	NA		
Agin et al., 2021 [16]	Prospective cohort	*H. pylori* (+)	199	13.47 ±4.49	197 (68)	0	0	NA	NA	NA	NA	NA	Histopathological examination of the gastric biopsy	There is a significant association between low serum vitamin D levels and the development of peptic ulcers. No significant association was detected between serum vitamin D levels and *H. pylori* infection
		*H. pylori* (-)	92			0	0	NA	NA	NA	NA	NA		
Rysbekov et al., 2024 [17]	Prospective cohort	*H. pylori* (+)	63	13 ±3.03	20 (30.8)	NA	NA	NA	NA	NA	NA	NA	Histopathological examination of the gastric biopsy and instant urease test	The study indicated that lower vitamin D levels (<20 μg/L) could be a predisposing factor for *H. pylori* infection in paediatric patients
		*H. pylori* (-)	65	12 ±3.03	29 (46.0)	NA	NA	NA	NA	NA	NA	NA		
Kuang et al., 2022 [18]	Cross-sectional	*H. pylori* CagA+	2100	NA	1131 (53.85)	NA	NA	NA	NA	NA	NA	NA	Detection of H. pylori CagA antibody	The study stated a nonsignificant association between serum vitamin D levels and *H. pylori* CagA seropositivity in the general population. However, serum vitamin d levels were associated with H. pylori CagA seropositivity among different ethnicities and individuals born outside the US.
		*H. pylori* CagA-	1412	NA	747 (52.9)	NA	NA	NA	NA	NA	NA	NA		
Habbash et al., 2022 [19]	Retrospective cross-sectional	*H. pylori* (+)	111	NA	48 (55.8)	NA	NA	NA	NA	NA	NA	NA	Either upper GI tract endoscopy biopsy examination, urea breath testing, or both	There is a significant association between vitamin D deficiency and the development of *H. pylori* infection
		*H. pylori* (-)	89	NA	38 (44.2)	NA	NA	NA	NA	NA	NA	NA		
Liu et al., 2023 [20]	Retrospective cohort	*H. pylori *(+)	415	47.3 ±12.8	194 (46.75)	0	0	NA	0	289 (69.64)	112 (26.99)	14 (3.37)	13 C-urea breath test	The study indicated that lower Vitamin D levels could be a risk factor for *H. pylori* infection
		*H. pylori* (-)	257	48.5 ±10.1	127 (49.42)	0	0	NA	0	168 (65.37)	70 (27.24)	19 (7.39)		
Askar et al., 2022 [21]	Cross-sectional	*H. pylori* (+)	644	NA	334 (51.86)	NA	NA	NA	NA	NA	NA	NA	H.pylori stool antigen test	There is a significant relationship between vitamin D deficiency and the development of *H. pylori *infection in children aged six to 18 years
		*H. pylori* (-)	956	NA	586 (61.29)	NA	NA	NA	NA	NA	NA	NA		
Assaad et al., 2019 [10]	Cross-sectional	*H. pylori* (+)	225	39.28 ±13.9	88 (39.1)	NA	NA	NA	0	109 (48.44)	116 (51.56)		Histopathological examination of the gastric biopsy	SNPs in TLR4 were inversely associated with vitamin D serum levels, which were negatively related to *H. pylori* infection
		*H. pylori *(-)	235	41.86 ±14.26	80 (34)	NA	NA	NA	0	128 (54.47)	107(45.53)			
Shafrir et al., 2021 [22]	Retrospective cohort	*H. pylori* (+)	75640	40.95 ±14.72	38576 (51)	NA	2663 (3.52)	26.89 ±5.64	NA	NA	NA	NA	90% Urea breath test (UBT) and the rest done by H. pylori stool antigen tests	The study indicated that lower vitamin D levels are associated with *H. pylori* infection
		*H. pylori* (-)	74,843	42.15 ±16.65	37422 (50)	NA	3465 (4.63)	26.45 ±5.62	NA	NA	NA	NA		
Korkmaz et al., 2015 [23]	Prospective cohort	*H. pylori* (+)	43	30.58±8.79	23 (53.49)	0	0	25.2±4.2	NA	NA	NA	NA	Histopathological examination of gastric biopsy and rapid urease test	The incidence of *H. pylori *infection was high in patients with vitamin D insufficiency
Surmeli et al., 2018 [24]	Cross-sectional study	*H. pylori* (+)	43	74.27 ±6.06	11 (25.6)	7 (16.3)	NA	NA	0	14 (32.6)	18 (41.9)	8 (18.6)	Histopathological examination of the gastric biopsy	The study indicated that lower Vitamin D levels could be a risk factor for* H. pylori* infection
		*H. pylori* (-)	211	77.53 ±7.76	91 (43.1)	52 (24.6)	NA	NA	2 (1)	86 (40.8)	79 (37.4)	24 (11.4)		
Chen et al., 2016 [25]	Survey	Metabolic syndrome (+)^a^	557	59.9±12.2	220 (39.49)	NA	NA	27.3 ±3.3	NA	NA	NA	NA	Urea breath test	The study found no significant difference regarding serum vitamin D levels in patients with and without *H. pylori* infection. However, the coexistence of* H. pylori* infection and vitamin D deficiency may increase the risk of developing metabolic syndrome
		Metabolic syndrome (-)^b^	1556	55.1 ±13.1	572 (36.76)	NA	NA	23.8 ±3.3	NA	NA	NA	NA		
Martins et al., 2018 [26]	Cross-sectional	*H. pylori* (+)	117	51.38 ±15.22	NA	NA	NA	NA	NA	NA	NA	NA	Histopathological examination of gastric biopsy	BsmI polymorphism could have a possible association with *H. pylori* infection
		*H. pylori* (-)	91	55.18 ±14.98	NA	NA	NA	NA	NA	NA	NA	NA		
Mihalache et al., 2016 [27]	Cross-sectional	*H. pylori *(+)	47	NA	NA	0	NA	44.04 ±5.992	0	0	0	47 (100)	Histopathological examination of gastric biopsy	The level of vitamin D was higher in *H. pylori*-infected patients with no statistical significance
		*H. pylori* (-)	46	NA	NA	0	NA	44.27 ±6.927	0	0	0	46 (100)		
Gao et al., 2020 [28]	Cross-sectional	*H. pylori* (+)	2113	1.0475 ±0.693	1,202 (30.9)	0	0	NA	NA	NA	NA	NA	H. pylori serum antibody test	There is a significant association between vitamin D deficiency and the development of *H. pylori* infection in children aged six to 36 months
		*H. pylori* (-)	4783	1.09 ±0.785	2685 (69.1)	0	0	NA	NA	NA	NA	NA		
Mohamed et al., 2020 [29]	Cross-sectional	*H. pylori *(+)	127	39.47 ±8.77	NA	NA	NA	NA	NA	NA	NA	NA	Histopathological examination of the gastric biopsy	The FokI and Apal VDR polymorphism could have a possible association with *H. pylori *infection
		*H. pylori* (-)	97	40.14 ±8.02	NA	NA	NA	NA	NA	NA	NA	NA		
Nasri et al., 2007 [30]	Cross-sectional	*H. pylori* (+)	36	47 ±17	21 (58.33)	36 (100)	NA	22 ±4.4	NA	NA	NA	NA	Serum H. pylori-specific IgG antibody	There is a significant association between serum vitamin D levels and the development of *H. pylori *infection
Han et al., 2019 [31]	Prospective cohort	*H. pylori* (+)	496	47.1 ±12.6	236 (47.58)	NA	NA	NA	NA	NA	NA	NA	Urea breath test	The study stated that serum vitamin D levels may affect *H. pylori *infection
		*H. pylori* (-)	257	48.1 ±10.2	127 (49.42)	NA	NA	NA	NA	NA	NA	NA		
Gerig et al., 2013 [32]	Retrospective cohort	*H. pylori* (+)	85	42.3 ±10.0	21 (24.71)	NA	NA	45.2 ±6.0	NA	NA	NA	NA	Histopathological examination of the gastric biopsy	The study stated no significant difference in serum vitamin D levels between the two groups
		*H. pylori* (-)	319	40.9 ±11.8	85 (26.65)	NA	NA	45.9 ±8.8	NA	NA	NA	NA		
Sorokman et al., 2020 [33]	Retrospective cohort	*H. pylori* (+)	86	NA	NA	0	NA	NA	NA	NA	NA	NA	Histopathological examination of the gastric biopsy	The study stated significantly lower serum vitamin D levels in patients with *H. pylori *infection

Outcomes

Serum vitamin D level: Seven studies found that the serum vitamin D level was statistically significantly lower in cases of positive *H. pylori *infection (P < 0.05) [10,20,22,24,28,31,33]. Only one study found a statistically significantly higher level of serum vitamin D in the positive *H. pylori* infection cases compared to the negative cases (P < 0.05) [17]. In the study by Chen et al. (2016), the study evaluated the associations between *H. pylori* infection, serum vitamin D level, and metabolic syndrome (MS). The study stated a significant association between vitamin D deficiency and MS (p < 0.05). However, they found no significant association between serum vitamin D and *H. pylori* infection (p > 0.05) [25]. In the study by Agin et al. (2021), they evaluated serum vitamin D levels in patients with peptic ulcers and patients without peptic ulcers, and they found that serum vitamin D levels were higher in people without peptic ulcers. However, *H. pylori* infection was more prevalent in patients with vitamin D deficiency [16]. Kuang et al. (2022) [18] evaluated the relationship between vitamin D and *H. pylori* CagA. The study found no significant association between serum vitamin D and CagA (p > 0.05); therefore, *H. pylori* infection. Moreover, Gerig et al. (2013) stated no statistically significant correlation between the *H. pylori *infection and serum vitamin D level [32]. This finding was also reported in the study by Mihalache et al. (2016). The study was conducted on obese patients with and without *H. pylori* and found no significant correlation between *H. pylori* infection and serum vitamin D level [27]. In the study conducted by Bahsi et al. in 2020 on sarcopenic patients, the study found that *H. pylori* in sarcopenic patients may lead to serum vitamin D deficiency ( Table 2) [10, 15-17, 19-24, 27-28, 30-33].

**Table 2:** Serum vitamin D level in the included population M ±SD: mean ±standard deviation; vitamin D level <20 μg/L is considered insufficient or inadequate for most people for bone health and overall health. NA: not available

ID	Study groups	Number	Serum vitamin D level, M ±SD	Serum vitamin D level < 20 μg/L	Serum vitamin D level ≥ 20 μg/L
						Bahsi et al., 2020 [15]	*H. pylori* (+)	32	24.31 ± 13.77	21 (65.6)	11 (34.38)
							*H. pylori* (-)	69	29.4 ± 14.59	32 (46.38)	37 (53.6)
Agin et al., 2021 [16]	*H. pylori* (+)	141	NA	85 (60.3)	56 (39.7)
	*H. pylori* (-)	150		36 (24)	114 (76)
Rysbekov et al., 2024 [17]	*H. pylori* (+)	63	17.57 ± 9.41	35 (55.55)	28 (44.44)
	*H. pylori* (-)	65	14.63 ± 6.9	51 (78.46)	14 (21.5)
Habbash et al., 2022 [19]	*H. pylori* (+)	61	NA	49 (80.33)	12 (19.67)
	*H. pylori* (-)	56		28 (50)	28 (50)
Liu et al., 2023 [20]	*H. pylori* (+)	415	16.7 ± 6.6	311 (74.94)	104 (25.06)
	*H. pylori* (-)	257	19.2 ± 8.0	157 (61.09)	100 (38.9)
Askar et al., 2022 [21]	*H. pylori* (+)	644	NA	282 (43.79)	362 (56.2)
	*H. pylori* (-)	956		311 (32.53)	645 (67.47)
Assaad et al., 2019 [10]	*H. pylori* (+)	225	18.04 ± 7.16	56 (24.89)	148 (65.78)
	*H. pylori* (-)	235	30.74 ± 15.66	109 (46.39)	119 (50.64)
Shafrir et al., 2021 [22]	*H. pylori* (+)	75,640	18.6 ± 9.8	NA	NA
	*H. pylori* (-)	74,843	20.1 ± 9.4		
Korkmaz et al., 2015 [23]	*H. pylori* (+)	43	14.7 ± 8.5	NA	NA
						Surmeli et al., 2018 [24]	*H. pylori* (+)	43	10.63 ± 8.67	37 (86.04)	6 (13.9)
*H. pylori* (-)	211	14.6 ± 11.35	142 (67.3)	69 (32.7)		
Mihalache et al., 2016 [27]	*H. pylori* (+)	47	17.25 ± 10.92	NA	NA
	*H. pylori* (-)	46	14.7 ± 7.72		
Gao et al., 2020 [28]	*H. pylori* (+)	2,113	25.2 ± 5.27	437 (20.68)	1676 (79.32)
	*H. pylori* (-)	4,783	25.93 ± 4.52	578 (12.08)	4205 (87.92)
Nasri et al., 2007 [30]	*H. pylori* (+)	36	10.5 ± 18.7	NA	NA
						Han et al., 2019 [31]	*H. pylori* (+)	496	17 ± 6.9	NA	NA
*H. pylori* (-)	257	19.2 ± 8									
Gerig et al., 2013 [32]	*H. pylori* (+)	81	49 ± 30	NA	NA
	*H. pylori* (-)	315	52 ± 29		
Sorokman et al., 2020 [33]	*H. pylori* (+)	86	15.4 ± 1.1	60 (69.77)	26 (30.23)
	*H. pylori* (-)	42	25.3 ± 1.4	15 (35.7)	27 (64.29)

Association between *H. pylori* and the level of vitamin D, either < or ≥ 20 ug/L: Regarding the association between *H. pylori* and vitamin D deficiency, seven studies showed that the frequency of vitamin D deficiency (<20 ug/L) was higher in the *H. pylori*-positive cases than in the negative cases [16,19-21,24,28,33]. Also, Bahsi et al. in 2020 [15] reported that *H. pylori *positivity increased the odds of vitamin D deficiency in sarcopenic patients. However, in two studies, the decline of serum vitamin D below 20 ug/L was higher in the negative cases of *H. pylori* compared to the positive cases [10,17]. In cases where the serum vitamin D level was ≥ 20 μg/L, eight studies reported that the level of serum vitamin D ≥ 20 μg/L was more common in negative *H. pylori* cases [15,16,19-21,24,28,33]. Only two studies reported that the association of the level of serum vitamin ≥ 20 μg/L was more common in positive cases of *H. pylori *[10,17]. However, Chen et al. (2016) found no significant difference regarding vitamin D serum levels in patients with and without *H. pylori* infection (Table 2) [25].

Genetic polymorphism and *H. pylori* infection: On assessing the relation between vitamin D genetic polymorphisms and *H. pylori* infection, three studies reported a significant association [10,26,29]. The studies collectively highlight that polymorphisms in the VDR gene, particularly rs2228570 (FokI), rs7975232 (ApaI), and rs1544410 (BsmI), as well as the TLR4 gene (rs4986790 and rs4986791), are significantly associated with *H. pylori* infection and vitamin D status. In Assad et al. (2019), the study reported that the TLR4 polymorphisms rs4986790G>A and rs4986791T>C were linked to vitamin D levels, which in turn influenced susceptibility to *H. pylori* infection, suggesting these SNPs may exert an indirect effect. Notably, individuals infected with *H. pylori *exhibited vitamin D deficiency, and their white blood cells expressed TLR4 at levels three times lower than those of their uninfected counterparts [10]. In the study by Martins et al. (2018), FokI, ApaI, and TaqI polymorphisms of the VDR gene did not show any difference between *H. pylori*-positive and *H. pylori*-negative groups. However, rs1544410 (BsmI) was the only VDR SNP that showed a significant association with *H. pylori*-positive individuals [26]. In the study by Mohamed et al. (2020), statistically significant differences were observed between *H. pylori*-infected and non-infected groups in the distribution of VDR gene polymorphisms. Regarding the rs7975232 variant, the CC genotype was more noticed among *H. pylori*-negative individuals, whereas the AC and AA genotypes were more common in the infected group. Similarly, for the rs2228570 variant, the TT genotype predominated in the non-infected group, while CT and CC genotypes were more frequently seen in those infected. The A allele of rs7975232 was significantly associated with *H. pylori *infection, in contrast to the C allele, which was more common among uninfected individuals. Likewise, the C allele of rs2228570 showed a higher frequency in the infected group, whereas the T allele was significantly enriched in the non-infected group [29].

Correlation between *H. pylori* CagA seropositivity and serum vitamin D levels: *H. pylori* CagA seropositivity refers to the presence of antibodies in the blood against the CagA protein, a virulence factor produced by certain strains of *H. pylori*. Two studies have reported the relation between *H. pylori* CagA seropositivity and vitamin D [18,33]. Kuang et al. (2022) [18] reported that there was no significant association between serum vitamin D levels and *H. pylori* CagA seropositivity in the general population. After adjusting for confounders, vitamin D levels were found to be positively associated with CagA seropositivity among different ethnicities and individuals born outside the US. However, in Sorokman et al. 2020, the study found that CagA-seropositive children had the lowest levels of serum vitamin D, averaging 12.1 (0.9) ng/mL, which was significantly lower than in CagA-negative children [33].

Quality assessment

The quality assessment of the included studies is shown in Table 3. Using the NOS, we assessed seven studies as having high quality [10,18,19,24,25,27,28] and 13 studies as having moderate quality [15-17,20-23,26,29-33].

**Table 3:** Quality assessment of eligible studies, as assessed by the Newcastle-Ottawa Scale Reference: [14]

Study ID	Selection	Comparability	Outcome	Total score
Liu et al., 2023 [20]	★★★☆	★☆	★★★	7
Agin et al., 2021 [16]	★★★☆	★☆	★★★	7
Rysbekov et al., 2024 [17]	★★★☆	☆☆	★★★	6
Shafrir et al., 2021 [22]	★★★☆	★☆	★★★	7
Korkmaz et al., 2015 [23]	★☆★☆	☆☆	★★☆	4
Han et al. 2019 [31]	★★★☆	★☆	★★★	7
Gerig et al., 2013 [32]	☆★★☆	★★	★★★	7
Sorokman et al., 2020 [33]	★★★☆	☆☆	★★★	6
Askar et al., 2022 [21]	★☆★★★	☆☆	★★☆	6
Assaad et al., 2019 [10]	★☆★★★	★★	★★★	9
Bahsi et al., 2020 [15]	★☆★★★	☆☆	★★★	7
Kuang et al., 2022 [18]	★★★★★	★☆	★★★	9
Habbash et al., 2022 [19]	★★☆★★	★★	★★★	9
Surmeli et al., 2018 [24]	★☆★★★	★☆	★★★	8
Chen et al., 2016 [25]	★★★★★	☆☆	★★★	8
Martins et al. 2018 [26]	★☆★★★	☆☆	★★★	7
Mihalache et al., 2016 [27]	☆☆★★★	★★	★★★	8
Gao et al., 2020 [28]	★★★★★	★☆	★★★	9
Mohamed et al., 2020 [29]	★☆★★★	☆☆	★★★	7
Nasri et al., 2007 [30]	☆☆★★★	☆☆	★★★	6

Discussion

This systematic review and meta-analysis synthesized evidence from 20 observational studies that examined the association between serum vitamin D levels and *H. pylori* infection. The results of our study underscore a potentially bidirectional interplay between vitamin D status and *H. pylori* pathogenesis, suggesting that vitamin D may serve not only as a biomarker of disease susceptibility but also as a modifiable factor influencing treatment outcomes.

Our study results reflect a consistent trend of reduced serum vitamin D levels among *H. pylori*-positive individuals across multiple geographic populations and age cohorts. While certain studies, such as Kuang et al. [18] and Chen et al. [25], reported non-significant associations, the overall effect size observed across 11 studies provides robust support for an inverse relationship. Additionally, the majority of the included studies showed an increased risk of serum vitamin D levels falling below 20 µg/L among *H. pylori*-infected individuals. This threshold is clinically relevant, as serum vitamin D levels <20 µg/L have been associated with impaired immune responses and increased susceptibility to bacterial infections [34]. This level was used by Chen et al., who documented successful eradication of *H. pylori* with increased serum vitamin D level equal to or above 20 µg/L, which provides a promising result of continuous increase in the serum vitamin level with time [25].

In addition to the quantitative serum analysis, we targeted the genetic analyses from three included studies to highlight key polymorphisms in the VDR and TLR4 genes that appear to modulate host susceptibility to *H. pylori*. Variants such as BsmI, ApaI, and FokI were significantly associated with *H. pylori* status, indicating a potential gene-environment interaction wherein host genetic predisposition amplifies the impact of hypovitaminosis D on infection risk. The findings add to the expanding evidence that vitamin D's immunomodulatory action mediated through the VDR plays a role in enhancing gastrointestinal mucosal immunity and modulating inflammatory signaling pathways.

Regarding the relationship between vitamin D levels and *H. pylori* virulence factors such as CagA, it remains inconclusive. We found controversial findings across the studies reporting this outcome. While Sorokman et al. observed significantly lower serum vitamin D levels among CagA-seropositive children [33]. Kuang et al. reported no significant association in general populations after adjustment for confounders [18]. This discrepancy may stem from variations in study design, population demographics, or assay sensitivity, highlighting the need for further research into the potential role of vitamin D in modulating the host response to distinct *H. pylori* strains.

The biological plausibility of our findings is supported by multiple proposed mechanisms through which vitamin D influences host defense against *H. pylori*. Vitamin D enhances expression of antimicrobial peptides such as cathelicidin and β-defensin, which disrupt bacterial membranes and suppress microbial colonization [35]. Moreover, activation of VDR in gastric epithelial cells has been shown to modulate autophagy and cytokine secretion, reducing gastric inflammation and facilitating bacterial clearance [36-38].

Emerging evidence also suggests that vitamin D may preserve gastric mucosal integrity through its role in epithelial barrier maintenance and oxidative stress reduction [39]. These pathways are particularly relevant given the chronic inflammation caused by *H. pylori* infection. Thus, vitamin D sufficiency may provide a protective immunological and epithelial milieu, reducing both initial susceptibility to infection and subsequent disease progression.

An updated systematic review and meta-analysis by Albatineh et al. in 2023 reported that serum vitamin D levels were significantly lower in infected cases with *H. pylori* compared to healthy controls. Also, the infected cases that were successfully managed and achieved complete eradication showed a higher level of serum vitamin D compared to those that did not achieve complete eradication [11]. The results of this previous systematic review align with our results. Also, Cai et al. evaluated the overall vitamin levels across infected, non-infected, completely eradicated, and non-eradicated *H. pylori* patients. The study agreed with our findings on the finding that serum vitamin D level was higher in non-infected cases compared to the infected individuals. Also, patients with complete eradication showed a significantly higher level of serum vitamin D compared to those with unsuccessful eradication [40]. Additionally, in the study conducted by Banama et al. in 2022, the study found that the addition of vitamin D supplementation to *H. pylori* eradication improved the eradication rate significantly (p < 0.05), showing promising results of serum vitamin D as a modifiable factor that would improve *H. pylori* treatment strategy [41]. Finally, a recent study done by Khan et al. in 2025 found that a deficiency in vitamin D could be a modifiable risk factor related to the development of *H. pylori* infections. These results highlight the necessity for additional long-term and interventional research to assess the possible benefits of vitamin D supplementation in preventing or managing *H. pylori* infections [42].

Limitations

Several limitations should be acknowledged. First, substantial heterogeneity was observed across studies (I² > 90%), likely reflecting differences in study design, diagnostic criteria for *H. pylori*, vitamin D assay methods, and population characteristics. Due to the substantial heterogeneity found across the studies, we adopted the use of a systematic review only without meta-analysis. Second, causality cannot be inferred from the included cross-sectional studies; it remains unclear whether vitamin D deficiency predisposes to *H. pylori *infection or vice versa. Longitudinal cohort studies and interventional trials are needed to elucidate temporal dynamics and therapeutic efficacy.

Third, variability in vitamin D thresholds and assay standardization presents a challenge. Definitions of deficiency ranged from <20 ng/mL to <30 ng/mL, and methods for measuring serum vitamin D (e.g., such as enzyme-linked immunosorbent assay (ELISA) and liquid chromatography-tandem mass spectrometry (LC-MS/MS)) may impact inter-study comparability. Future studies should adopt standardized protocols for vitamin D measurement and *H. pylori* diagnosis to enhance reproducibility and comparability.

Additionally, genetic analyses were limited to three studies, with varying degrees of statistical power. Although the associations reported are promising, replication in larger multiethnic cohorts and functional assays is required to validate these findings and elucidate their mechanistic significance.

Implications and Future Recommendations

Our findings carry a significant impact on *H. pylori* management and prevention. The vitamin D factor would serve as an adjunct in risk stratification and treatment strategy in settings with a high burden of *H. pylori* and prevalent vitamin D deficiency, such as low- and middle-income countries. Identifying individuals with concurrent *H. pylori* infection and vitamin D deficiency may allow for tailored interventions, including nutritional supplementation and lifestyle modifications.

Moreover, future randomized controlled trials should evaluate the efficacy of vitamin D supplementation as an adjunct to standard triple or quadruple therapy for *H. pylori* eradication. If proven beneficial, such interventions could be particularly valuable in populations with limited access to healthcare resources, where eradication failure rates remain high.

## Conclusions

In conclusion, this systematic review and meta-analysis resulted in a significant inverse association between serum vitamin D levels and *H. pylori* infection, with implications for pathogenesis, eradication success, and public health interventions. However, we could not confirm causality; our findings provide a strong base for further research into vitamin D’s role in gastric immunity and its utility as a modifiable risk factor in managing *H. pylori *infection.

Integrating epidemiological evidence with molecular findings could facilitate future research in developing more targeted and effective strategies for the prevention and treatment of *H. pylori* infection.
